# Electrochemical DNA Sensor Based on Acridine Yellow Adsorbed on Glassy Carbon Electrode

**DOI:** 10.3390/s21227763

**Published:** 2021-11-22

**Authors:** Tatjana Kulikova, Anna Porfireva, Alexey Rogov, Gennady Evtugyn

**Affiliations:** 1 A.M. Butlerov’ Chemistry Institute, Kazan Federal University, 18 Kremlevskaya Street, 420008 Kazan, Russia; wefy2009@yandex.ru (T.K.); porifreva-a@inbox.ru (A.P.); 2 Interdisciplinary Center of Analytical Microscopy, Kazan Federal University, 18 Kremlevskaya Street, 420008 Kazan, Russia; alexeyrogov111@gmail.com; 3 Analytical Chemistry Department, Chemical Technology Institute, Ural Federal University, 19 Mira Street, 620002 Ekaterinburg, Russia

**Keywords:** electrochemical DNA sensor, doxorubicin determination, carbon black, cyclic voltammetry, drug determination

## Abstract

Electrochemical DNA sensors offer unique opportunities for the sensitive detection of specific DNA interactions. In this work, a voltametric DNA sensor is proposed on the base of glassy carbon electrode modified with carbon black, adsorbed acridine yellow and DNA for highly sensitive determination of doxorubicin antitumor drug. The signal recorded by cyclic voltammetry was attributed to irreversible oxidation of the dye. Its value was altered by aggregation of the hydrophobic dye molecules on the carbon black particles. DNA molecules promote disaggregation of the dye and increased the signal. This effect was partially suppressed by doxorubicin compensate for the charge of DNA in the intercalation. Sensitivity of the signal toward DNA and doxorubicin was additionally increased by treatment of the layer with dimethylformamide. In optimal conditions, the linear range of doxorubicin concentrations determined was 0.1 pM–1.0 nM, and the detection limit was 0.07 pM. No influence of sulfonamide medicines and plasma electrolytes on the doxorubicin determination was shown. The DNA sensor was tested on two medications (doxorubicin-TEVA and doxorubicin-LANS) and showed recoveries of 102–105%. The DNA sensor developed can find applications in the determination of drug residues in blood and for the pharmacokinetics studies.

## 1. Introduction

DNA sensors have found increasing attention in recent decades due to the variety of opportunities they offer in molecular biology, pharmacy, diagnostics of pathogenic bacteria and viruses and carcinogen monitoring [1,2,3,4]. Although most commercialized applications of DNA sensors utilize detection of hybridization events between the DNA probe and a biological target associated with particular genes [5,6], the determination of small molecules able to specifically interact with the DNA molecules has become important. Such DNA sensors make it possible to determine antitumor drugs [7,8,9], DNA damaging factors [10,11,12] and other biomolecules affecting steric DNA structure [13]. Such biosensors can be used for the screening of new drugs, investigation of pharmacokinetics of existing drugs, establishment of potential hazards related to the DNA damage [14] and protecting effect of antioxidants preventing such damage caused by reactive oxygen species [15].

Contrary to rather bulky proteins and DNA sequences, small molecules, being bonded to the DNA receptor, insignificantly alter the conditions on the transducer interface. This makes impossible their reliable detection and decreases the sensitivity of appropriate measurement. Electrochemical detection principles offer many opportunities to solve the problems of the response by the implementation of catalytic redox conversion of redox labels/indicators and recording changes in the conditions of the electron transfer caused by DNA interactions. Thus, target binding of an analyte can change the oxidation currents attributed to certain nucleobases, mostly guanine, in the DNA sequence [14]. In the case of the oxidative DNA damage, an 8-oxoguanince oxidation peak appears on a voltammogram. In other measurement modes, redox active labels and diffusionally free redox indicators have been also utilized for an analyte detection. Changes in their signals are caused by electrostatic interactions or steric hindrance of the electron transfer resulted from the NDA—analyte interaction. Methylene blue and ferrocene are mostly used in such investigations for DNA labeling and [Fe(CN)_6_]^3−/4−^ and [Ru(NH_3_)_6_]^3+^ ions as soluble redox indicators [16,17,18,19,20,21]. Their use showed high sensitivity of the response. However, the measurement protocol is complicated, with the necessity of covalent attachment of labels and non-specific signal changes related to the adsorption of the redox indicators on the electrode. 

Redox active polymers are considered as a promising alternative to the design of label-free DNA-sensors [22,23]. In them, biospecific interactions with DNA alter the intrinsic redox activity of underlying polymer film due to variation of the charge distribution and/or shift of the redox equilibrium within the layer. Polyaniline is the most known polymer used for electric wiring of biomolecules [24,25]. DNA is also applied as a template to facilitate the formation of polyaniline in chemical and electrochemical oxidation. Polypyrrole, polythiophene and their derivatives have been mostly utilized for anchoring DNA oligonucleotides and labels [26,27,28,29]. Being rather sensitive in the detection of hybridization events, they have not found broad applications in small molecules determination.

Polymeric forms of phenothiazine and phenazine dyes, e.g., methylene blue [30,31], thionine [32], methylene green [33] and neutral red [34,35], offer opportunities for improving the performance of electrochemical sensors due to their electrocatalytic activity and easy deposition on the electrode by means of repeated cycling of the potential. However, their application in the assembly of DNA sensors is limited mostly by hybridization detection and anchoring other redox labels. Recently, we have shown the possibility to assemble DNA sensors based on the electropolymerization of Azure A and Azure B followed by electrostatic adsorption of native DNA [36,37,38]. The DNA sensors were successfully applied for sensitive determination of anthracycline. Nevertheless, the efficiency of electropolymerization was lower than that of above-mentioned phenothiazines and the obtained films were thin enough to limit both the accuracy of the measurement and biosensor lifetime. Low solubility in aqueous media and aggregation liability are considered as probable reasons of these limitations. 

In this work, we report on electrochemical performance of acridine yellow (AY) within the surface layer of carbon black (CB) as transducer of the DNA sensor. To the best of our knowledge, this is the first example of the application of acridine dyes specifically interacting with DNA on the electrode without polymerization and implementation in inert films (Web of Science, search on “acridine yellow” + “DNA” + “sensor”). The modification protocol offers reliable and sensitive determination of DNA adsorbed as well as its damage and interaction with various species.

Acridine dyes are widely used in biomolecular and biochemical investigations due to specific interactions with DNA in combination with chemical stability and high fluorescence intensity [39]. They are applied for cell staining and cancer imaging and exert antitumor, antibiotic, antiviral and antifungal action. DNA intercalation with acridine dyes results in widening DNA helix followed by disturbing steric molecule structure and changes in spatial charge distribution [40]. Meanwhile, the electrochemical investigation of DNA–acridine dye interactions has been performed only in few publications. Thus, changes in electrochemical characteristics of catechol and acridine dyes in the presence of DNA were investigated with electrochemical and fluorescent biosensors [41]. The same authors reported sensitive determination of acridine dyes in urine with electrochemical DNA sensor based on glassy carbon electrode (GCE) and protecting poly(vinyl alcohol) membrane [42]. Both concentration and DNA intercalation ability of the dyes were quantified using peak currents on square wave voltammograms. In our approach, AY was adsorbed on the electrode surface and then covered with DNA. Changes in the redox activity of the surface layer were investigated in conditions preventing the AY electropolymerization to assess the influence of both DNA and its intercalator on the electrochemical characteristics of the biosensor.

## 2. Materials and Methods

### 2.1. Reagents

AY (3,6-diamino-2,7-dimetyhlacridine, dye content 90%), low-molecular DNA from salmon tests (liophylized powder, <5% protein, A_260/280_ 1.4), doxorubicin hydrochloride ((7S,9S)-7-[(2R,4S,5S,6S)-4-amino-5-hydroxy-6-methyloxan-2-yl]oxy-6,9,11-trihydroxy-9-(2-hydroxyacetyl)-4-methoxy-8,10-dihydro-7H-tetracene-5,12-dione, 98–102%), potassium hexacyanoferrate (III) (99%), potassium hexacyanoferrate (II) (98.5–102%) and N,N′-dimethylformamide (DMF) were purchased from Sigma-Aldrich, Dortmund, Germany, chitosan (mol. weight 100,000–30,000 D) from Acros Organics and CB (>99.95% C) from Imerys. All the working solutions were prepared using Millipore Q^®^ water (Simplicity^®^ water purification system, Merck-Millipore, Molsheim, France). Other reagents were of analytical grade. Electrochemical measurements were performed in 0.025 M phosphate buffer consisting of 24.7 mM Na_2_HPO_4_, 0.28 mM NaH_2_PO_4_ and 100 mM NaNO_3_. If necessary, the pH of the buffer was adjusted by adding HCl or NaOH.

### 2.2. Apparatus

Direct current linear sweep voltammetry was used for electrode characterization at room temperature using Autolab PGSTAT 302N (Metrohm Autolab b.v., Utrecht, The Netherlands) and potentiostat/galvanostat μSTAT 400 (Metrohm DropSens, Oviedo, Spain). All the measurements were performed in the 5 mL three-electrode cell equipped with GCE (ALS Co Ltd., Tokyo, Japan, Cat. No 012744, 0.0167 cm^2^) modified with CB, AY and DNA as the working electrode, Pt stripe (ALS Co Ltd., Cat. No 002233) as the auxiliary electrode and the Ag/AgCl/3 M KCl reference electrode (Metrohm Autolab Cat. No 6.0726.107). Electrochemical impedance spectroscopy (EIS) measurements were performed with the FRA 2 module of the same potentiostat. The potential frequency was varied from 0.04 Hz to 100 kHz, amplitude of sine potential was equal to 5 mV and equilibrium potential was calculated as a half sum of peak potentials on cyclic voltammograms recorded on the modified electrode in the equimolar mixture of 10 mM [Fe(CN)_6_]^3−/4−^ ions. The EIS parameters were calculated from the Nyquist diagram corresponded to the *R*(*RC*)(*RC*) equivalent circuit using the NOVA 1.11 software (Metrohm Autolab, Utrecht, The Netherlands). 

Scanning electron microscopy (SEM) images of the electrode coatings were obtained with the high-resolution field emission scanning electron microscope Merlin™ (Carl Zeiss).

### 2.3. GCE Modification and DNA Sensor Assembling

GCE was first mechanically polished using aluminum oxide powder (grain size 0.3 µm) (polishing set for solid-state electrodes, ALS Co Ltd., Cat. 6.2802.000) and cleaned with acetone and deionized water. After that, it was electrochemically cleaned by multiple cycling of the potential in 0.1 M H_2_SO_4_ until the background current stabilization. Then, the electrode was washed with deionized water and dried. CB suspension was prepared by 60 min sonication of the CB in chitosan (1.35 mg in 1 mL of 0.05 M HCl) or DMF (0.1 mg/mL). Then, 2 μL of the CB suspension was spread on the working area of the electrode. In case of the chitosan film, the electrode was additionally treated with 0.1 M NaOH. Then, the electrodes were dried at 60 °C for 40 min, and 2 μL of 1.0 μM AY solution in warm water (40–50 °C) were added onto the CB layer and dried again. In some experiments, AY was added to the CB suspension prior to its deposition on the electrode. The content of the modifier was varied by changing the aliquot volumes of the CB and the dye. DNA aliquot was added to the GCE modified with CB and AY either directly from its solution in phosphate buffer or after incubation of the DNA with doxorubicin solution.

## 3. Results

### 3.1. Electrochemical Properties of AY in Aqueous Solution

**Figure 1:**
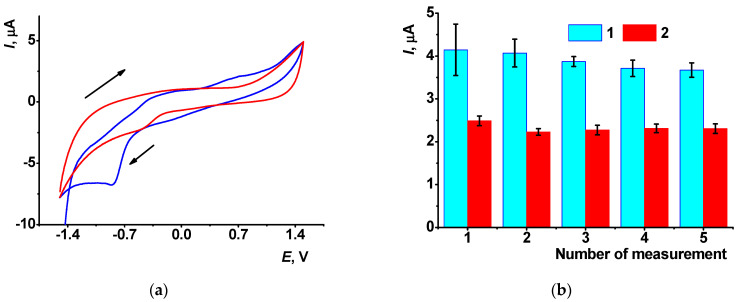
(**a**) Cyclic voltammogram of 1.0 μM AY in 0.025 M phosphate buffer containing 0.1 M NaNO_3_, pH = 7.0, on the bare GCE, 100 mV/s. Arrows indicate direction of the potential scamming. (**b**) Cathodic AY peak currents in the series of consecutive measurements on the same bare glassy carbon (1) and that covered with the CB suspension in chitosan (2). Average ± S.D. for six replications on individual electrodes.

Although the AY solubility was found to be insufficient for the full characterization of its activity in homogeneous conditions, preliminary experiments were performed with 1.0 μM AY dissolved in warm 0.025 M phosphate buffer after removal of the oxygen by nitrogen purging. On bare GCE, one irreversible cathodic peak was found at −0.85 V (Figure 1a), probably corresponding to the reduction of the aromatic core of the molecule. 

The cathodic peak current (*I_pc_*) linearly depended on the square root from the scan rate (ν^1/2^). Appropriate plots in the coordinates *I_pc_*–ν and *I_pc_*–ν^1/2^ are presented in Figure S1 (supplementary material). The slope of the plot in bi-logarithmic coordinates (Equation (1)) is near 0.5, indicating diffusional control of the electrode reaction [43].

log(*I_pc_*, μA) = (0.11 ± 0.16) + (0.54 ± 0.02)·log(ν, mV/s), *R*^2^ = 0.998, *n* = 8 (1)

In a series of consecutive records, the peak tended to decrease until stabilization to the third scan (Figure 1b). In the anodic area of the potentials, a number of poorly resolved small peaks could be seen. To increase the surface area and the currents recorded, it was proposed to cover the GCE with the CB deposited from its suspension in chitosan. Based on the ferricyanide cyclic voltammogram and the Randles–Sevcik equation, the ratio of effective and geometric surface area was equal to 1.8 for deposition of 2 μL of 1.35 mg/mL CB suspension in chitosan. 

The CB deposition did not alter the AY voltammograms in the cathodic area of the potentials. The cathodic peak current slightly decreased with the with maximum at pH = 4.0 (Figure S2 in supplementary material). However, its height was unexpectedly lower than that recorded on bare GCE. Meanwhile, the peak was more reproducible and did not drift in the series of consecutive records (Figure 1b). Changes in the peak currents can be attributed to aggregation of the AY molecules on the CB particles followed by partial shielding of the conductive area of the electrode.

**Figure 2:**
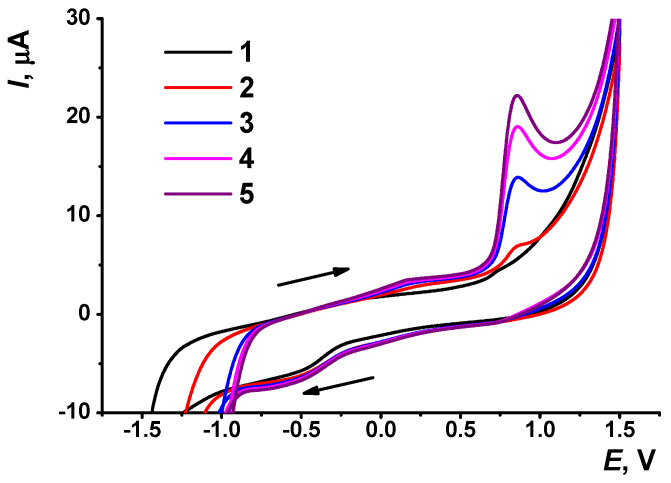
Cyclic voltammograms recorded on the GCE covered with CB (2 μL of 1.35 mg/mL in chitosan) and DNA (20 min incubation) in 1.0 μM AY solution in phosphate buffer, pH 4.0. The numbers (1–5) correspond to the number of potential scan performed after the immersion of the electrode in the dye solution. Arrows indicate the direction of the potential scan, 100 mV/s.

Acridine dyes interact with DNA molecules [40,41,42]. To prove this phenomenon for AY in the selected experimental conditions, the GCE covered with the CB layer was incubated in the DNA solution for 20 min. After that, cyclic voltammograms were recorded in the presence of the 1.0 μM AY solution as described above (Figure 2). At first cycle, the voltammogram recorded in such solution was similar to that obtained on the GCE, both bare and covered with the CB with no DNA. Starting from the second cycle of the potential, an irreversible anodic peak appeared and rapidly grew at 0.85 V. The peak potential was similar to that which corresponded to minor anodic peaks found on bare GCE in the same measurement conditions. Thus, interaction with the DNA promoted oxidation of the AY molecules. The AY anodic peak was quite reproducible, with a relative standard deviation (R.S.D.) of about 3.5% for three repetitions with individual electrodes. It retained on the voltammograms after the transfer of the electrode in the phosphate buffer with no AY, indicating surface-confined reactions on its surface. The cathodic AY peak was shifted to less negative potentials against measurements with no DNA. It transformed into the wave at about −0.40 V. Its height was much less sensitive to the measurement conditions and irregularly changed within 5% of absolute value.

### 3.2. Deposition of the AY and CB on the GCE

As the anodic AY signal was retained in phosphate buffer with no dye, interaction with DNA promoted the adsorption of the dye on the electrode. In the next step of the investigation, the AY was deposited on the CB layer of GCE. The appropriate surface layer is denoted as CB/AY. Anodic peak changes were monitored in the presence of DNA. Variation of the surface layer content was achieved by mixing stock solutions of the CB and of the dye in the volume ratio, varying from 1:1 to 10:1. Absolute concentrations of the components in the final mixture are presented in Table S1. In all the experiments, 2 μL aliquot was placed on the GCE working area. The modified electrodes were equalized in the working buffer and then cyclic voltammograms were recorded in the conditions specified for the homogeneous AY solution (Section 3.1). 

The morphology of cyclic voltammograms recorded on the GCE with the CB/AY layer was similar to that obtained in the AY solution on the GCE covered with CB in the presence of dissolved DNA (Figure S3 in supplementary material). The peak shape was typical for diffusionally controlled peaks, though no free dye was present in the solution. This means the adsorption of the AY molecules did not meet the Langmuir isotherm. Probably, the formation of the multilayers and nanoaggregates on the CB particles takes place on the GCE surface. In this case, the electron exchange between reduced and oxidized dye molecules adsorbed mimics their diffusion from the solution.

**Figure 3:**
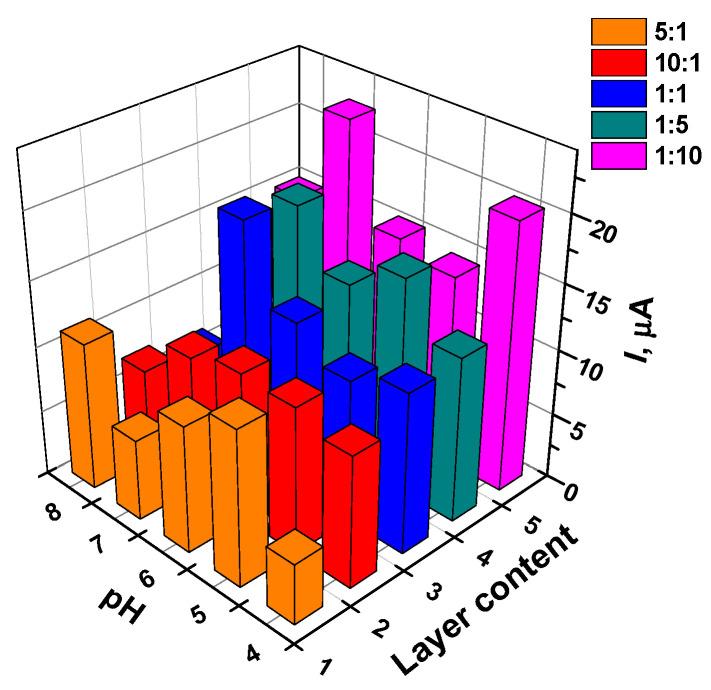
The dependence of the anodic peak current on the GCE covered with the mixture of the CB and AY on the surface layer content and the pH. The surface layer content is expressed as v:v ratio of the components mixed prior to deposition on bare GCE. 1—5:1, 2—10:1, 3—1:1, 4—1:5, 5—1:10 (the numbers also correspond to appropriate lines of Table S1).

The anodic AY peak current depended on the pH of the solution and on the layer content (Figure 3). For relatively low dye content (layers 5:1 and 1:1), the peaks obviously increased in the middle of the pH region, while the highest content of the dye exerted maximal signal at extreme pH values (pH = 8.0 and 4.0). The pH dependency reflects both different reactivity of the dye molecules in the electron transfer and importance of this parameter for the adsorption and aggregation of the dye on the electrode. Within the whole range of the layer contents, higher AY quantities resulted in bigger peaks on voltammograms.

**Figure 4:**
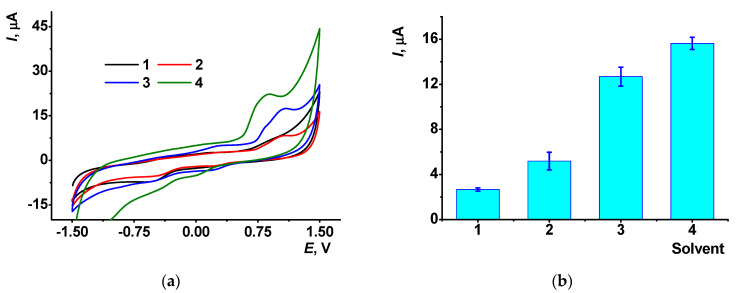
(**a**) Cyclic voltammograms recorded on the GCE covered with CB/AY (1:1 mixture, chitosan, 2 μL per electrode) after 10 min incubation of the electrode in organic solvent. (**b**) The AY anodic peak currents recorded after the contact of the GCE covered with 1:1 mixture of CB/AY in chitosan with DMF (1), ethanol (2), chloroform (3) and phosphate buffer, pH = 4.0 (4). Average from three repetitions with individual electrodes.

As aggregation affects the electrochemical performance of the AY in the surface layer, it was proposed to treat the CB/AY film with organic solvent to dissolve AY aggregates and change its distribution on the CB particles. The electrodes were treated with a small portion of organic solvent and then dried on air so that no losses of the dye were expected in the following contact of the electrode with the buffer. In these experiments, the AY oxidation peak decreased after the 10 min contact with DMF, ethanol or chloroform (Figure 4). 

### 3.3. The Influence of DMF on the Electrochemical Properties of the CB/AY Modified GCE with Adsorbed DNA

The experiments were continued with DMF exerting the maximal effect of the AY peak. Chitosan applied for the preparation of the CB suspension as a film-forming material was replaced with DMF to exclude partial shielding of the CB surface with hydrophilic polymer film. Such a replacement resulted in the stabilization of the CB suspension and in the formation of a dense and mechanically rigid film on the GCE surface. In the following experiments, the GCE covered with the CB/AY film obtained in the presence of DMF was incubated in the DNA solution for a certain period (15–40 min). Then, the electrode was dried and treated with additional DMF aliquot to disintegrate the AY aggregates. 

Contrary to the influence of DMF on the CB/AY layer with no DNA, such a protocol of the layer assembling resulted in about a twofold increase in the anodic AY peak on voltammograms. The slope of the dependence of the anodic peak current (*I_pa_*) on the scan rate (ν) in bi-logarithmic coordinates indicated mixed adsorption–diffusion control of the electron transfer (Equation (2)).

log(*I_pc_*, μA) = (−0.17 ± 0.10) + (0.77 ± 0.03)·log(ν, mV/s), *R*^2^ = 0.992, *n* = 6 (2)

**Figure 5:**
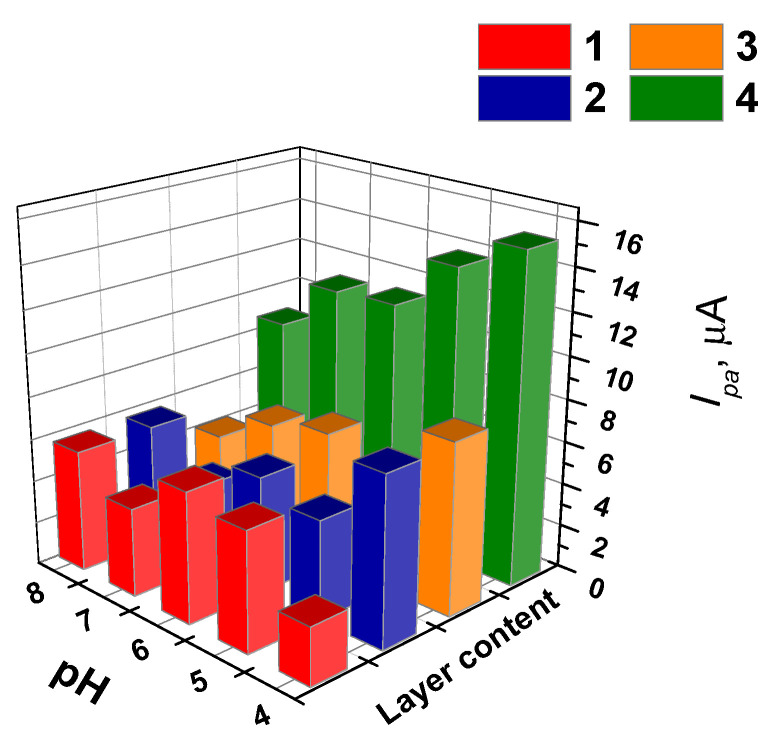
The dependence of the anodic AY peak current on the GCE covered with the CB/AY (1:1) and adsorbed DNA from 1 mg/mL solution (incubation 10 min) on the modification protocol and pH. 1,2—CB suspended in chitosan, 3,4—CB suspended in DMF; 1,3—DMF treatment after the DNA adsorption, 2.4–40 min incubation with DMF.

The comparison of the AY signals recorded with different protocols of the GCE modification is presented in Figure 5.

As could be seen, the use of DMF instead of chitosan increased the peak currents within the whole pH range. The AY peaks regularly increased with transfer from basic to acidic media in the presence of DMF and changed irregularly in the presence of chitosan. The latter fact can be explained by buffering properties of film-forming material that smooths the pH changes in the bulk solution. Post-treatment of the layer with DMF increased twofold the slope of the pH dependence of the peak current. This confirms the suggested mechanism of the influence of organic solvent on the aggregation of the dye and its distribution among the CB particles and DNA molecules within the layer. 

Higher amounts of the dye taken for modification resulted in a minor decrease in the anodic peak current due to coverage of the electrode surface with non-conductive dye layer. In this respect, DMF promoted redistribution of the AY molecules that increased their accessibility for the electron transfer. 

The DNA concentration in the range from 0.2 to 2 mg/mL applied on the stage of the electrode modification increased the AY current by a maximum of 75% (Figure S4 in supplementary material). The DNA influence monitored at a constant dye concentration increased up to maximum at 2 mg/mL. This concentration was then used in the following measurements. Higher DNA quantities decreased the reproducibility of the AY peaks on voltammograms and mechanical durability of the layer. In some cases, cracks appeared in the surface CB layer in the adsorption of the high DNA quantities and in the following DMF treatment of the layer. This made impossible DNA sensor operation.

The period of incubation in the DNA solution also affects the AY signal on voltammogram (Figure S5 in supplementary material). At short incubation periods, an increase in the DNA concentration slightly affected the anodic peak current. At 40 min incubation, the influence of DNA became the highest, and the concentration of 2 mg/mL resulted in a 30% increase in the signal. This might result from the rather slow process of disintegration of the dye aggregates caused by DNA. The parameters of the assembling of the surface layer were then used for the other experiments.

### 3.4. EIS and SEM Characterization 

**Figure 6:**
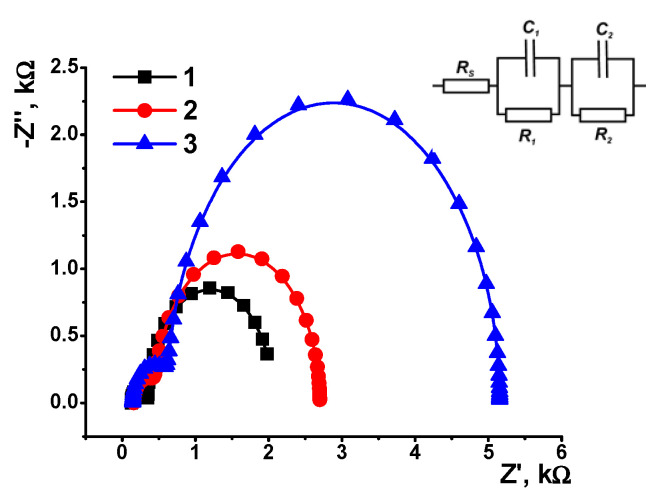
The Nyquist diagrams of the impedance spectra recorded on the GCE covered with CB (1), CB/AY (2) and that after adsorption of DNA (3). Frequency range from 0.04 Hz to 100 kHz, amplitude of the applied sine potential 5 mV, 0.025 M phosphate buffer, pH = 4.0. Inset: equivalent circuit applied for the data fitting. *R* is the charge transfer resistance and *C* constant phase element Rs is the electrolyte resistance. Index 1 corresponds to the solution–layer interface and index 2 to the electrode–layer interface.

Assembling the surfaced layer was monitoring using EIS measurements in the presence of an equimolar mixture of 0.01 M K_3_[Fe(CN)_6_] and 0.01 M K_4_[Fe(CN)_6_]. Figure 6 shows the Nyquist diagram. Two semicircles correspond to the electrode–layer and layer–solution interfaces. Radii of semi-circles recorded at high frequency correspond to the limit step of charge transfer. Introduction of AY and DNA increased the charger transfer resistance with a bigger effect on the outer interface. The EIS parameters were calculated by fitting the EIS data and are presented in Table 1. The exponent of the constant phase element (*n*) was near one in all the cases, indicating ideal capacitance behavior. 

The capacitance of the interfaces changed in the direction opposite that of the charge transfer resistance. This corresponds to the interaction of the oppositely charged species and the changes in the total charge of the interface. Indeed, cationic AY molecules interact with carboxylate groups on the surface of the CB particles and DNA with negative charge of phosphate groups of the backbone with the AY molecules. Disaggregation of the AY promotes the neutralization of the charge so that the permeability of the layer for negatively charged ferri/ferrocyanide ions decreased. Thus, deposition of individual components of the surface layer provides changes in its permeability and charge, indicated by the AY signals and EIS parameters.

**Figure 7:**
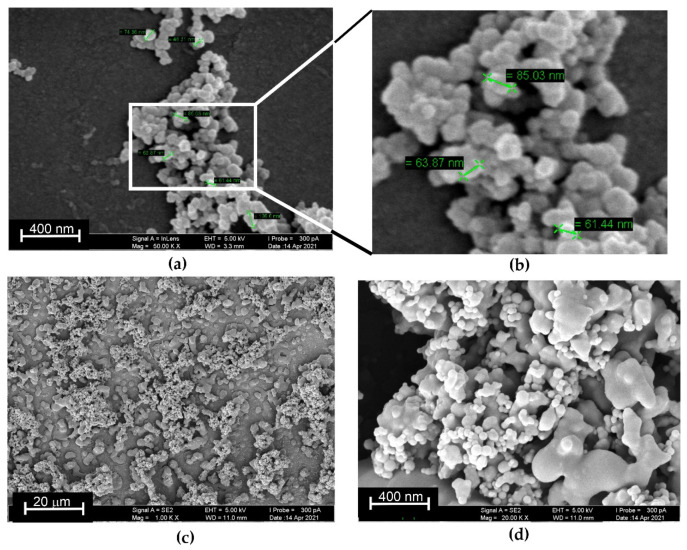
SEM images of the electrode surface covered with CB/AY and treated with DMF (**a**,**b**) and that preliminary treated with DNA solution and then additionally treated with DMF (**c**,**d**).

SEM images indicate the efficiency of DMF treatment on the AY aggregation on the electrode. From Figure 7, one can see the aggregates on the GCE covered with CB using DMF as film-forming material. The size of roundish particles varied from 40 to 79 nm. Additional treatment with DMF after the DNA adsorption changed the morphology of the surface layer. Together with small particles.

The size of roundish particles varied from 40 to 79 nm. Additional treatment with DMF after the DNA adsorption changed the morphology of the surface layer. Together with small particles, compact phase parts appeared, looking like a frozen melt. Thus, DNA presence stimulated the disturbance of the AY aggregates and increased the surface accessible for electron transfer to the CB and electrode.

### 3.5. Doxorubicin Determination

#### 3.5.1. Selection of Working Conditions of Doxorubicin Determination

The DNA adsorbed in the surface layer retains its ability for biospecific interactions. This was proved on the example of doxorubicin. This is an anthracycline drug used in cancer chemotherapy [44]. Due to its rather high cardiotoxicity [45], simple reliable sensors are demanded for doxorubicin determination. Additionally, doxorubicin is commonly used as a model in DNA intercalators in the development of electrochemical DNA sensors. Recently, the interest in the simple reliable electrochemical sensors for antibiotics determination has grown enormously, and some successfully utilize DNA receptors in their assembly [46,47].

**Figure 8:**
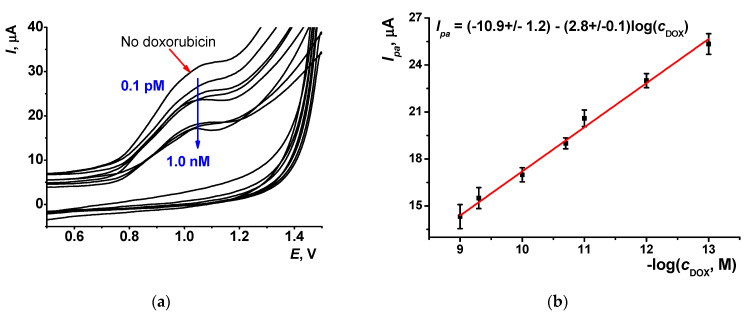
(**a**) Cyclic voltammograms recorded on the GCE covered with CB/AY (1:1 mixture, DMF, DNA, 2 μL/mL) after 20 min incubation in doxorubicin solution (0, 0.1, 1.0 pM, 0.01, 0.1, 1.0 and 5.0 nM). (**b**) Calibration plot of doxorubicin (DOX), measurements in 0.025 M phosphate buffer, pH = 3.0. Average for three individual electrodes.

Doxorubicin can intercalate native DNA by implementation between flat pairs of the nucleobases of the DNA helix. This reaction affects both the volume and charge distribution of the biopolymer. The experiments were performed by 20 min incubation of the GCE covered with the CB/AY, DNA and treated with DMF in the doxorubicin solution, followed by the record of the voltammogram. As expected, the contact with intercalator resulted in a decrease in the anodic AY peak. This can be attributed to lower efficiency of DNA in the disaggregation of the AY particles on the electrode. Figure 8 shows the voltammograms and calibration curve linear in the range from 0.1 pM to 1.0 nM. The limit of detection was calculated from the criterion 3·S.D./*b*, where S.D. is a standard deviation of the background current and *b* is the slope of calibration curve. It was found to be 0.07 pM. The slope of calibration curve decreased with the pH of incubation from 2.8 μA/log(C, M) at pH 3.0 to 1.2 μA/log(C, M) at pH 5.0. Higher pH does not allow reliable measurement of the peak shift.

An incubation period of 20 min was found to be optimal for measurements. Lower periods resulted in a lesser sensitivity of the signal, while longer incubation resulted in sufficient increase in the deviation.

The characteristics of doxorubicin determination were comparable or better than those of electrochemical sensors and DNA sensors described in the literature. The comparison of the analytical characteristics is presented in Table 2. 

In electrochemical sensors, doxorubicin oxidation with transfer of two electrons is mostly considered, and the proposed modifiers both decreased overvoltage of the electron transfer and the peak currents. In electrochemical DNA sensors, redox active polymers were utilized for both DNA accumulation and intercalation detection. Changes in the intrinsic redox activity of the polymers were recorded by cyclic voltammetry and EIS. The only exception is the monitoring of doxorubicin based on the current of guanine oxidation in the DNA molecule [56]. The DNA sensor showed remarkable durability and was successfully tested on clinical samples.

#### 3.5.2. Measurement Precision and DNA Sensor Lifetime

All the DNA sensors were used only once for doxorubicin measurement. The attempts of recovery of the biosensor after its contact with doxorubicin by washing with phosphate buffer were unsuccessful. In a series of repeated measurements with the same DNA sensor, the shifts of the peaks current additively increased with each contact with the doxorubicin solution. Meanwhile, a rather high deviation of repeated measurement did not allow recommending this protocol.

Repeatability of the signal to doxorubicin was calculated for five individual DNA sensors prepared with the same set of reagents. For 0.1 nM doxorubicin and 20 min incubation, R.S.D. was equal to 4.5% for freshly prepared biosensors and about 6% after three months stored in dry conditions. It should be also noted that the storage of the DNA sensor in dry conditions for six months resulted in decrease in the initial AY peak current with no respect of the DNA and doxorubicin constant. Nevertheless, relative shifts of the signal remained about the same within the whole storage period, although the R.S.D. increased to 10% on the 180th storage day. Taking into account both the absolute value and its decay, a DNA sensor life time of three months were established as providing reliable determination of intercalators.

#### 3.5.3. Selectivity and Real Sample Analysis

Selectivity of the signal to doxorubicin was tested using some other medicines in the same measurement protocol. As expected, other anthracycline drugs (daunorubicin, idarubicin) exerted similar changes in the AY signal. Thus, their total content could be determined. Sulfonamide preparations (on the example of sulfamethoxazole) affected the oxidation AY peak irregularly by 10–15% of the initial value starting from 0.1 μM. Their influence on the doxorubicin determination can be easily overcome by dilution of the sample.

In a similar manner, the influence of bovine serum albumin (as a model of serum proteins) and of plasma electrolytes (0.45 g NaCl, 0.021 g KCl, 0.016 g CaCl_2_·2H_2_O, 0.005 g NaHCO_3_, 0.015 g of MgSO_4_, and 0.025 g of NaH_2_PO_4_·2H_2_O per 50 mL of water [58]) was estimated. The recoveries of 110 ± 7% and 102 ± 6% were found for 0.1 nM doxorubicin.

The developed DNA sensor was tested on the determination of doxorubicin in commercial medications Doxorubicin-TEVA^®^ and Doxorubicin-LANS^®^ (lyophilizates for intravascular injection solutions) purchased at a local pharmacy market. Samples were dissolved in 0.025 M phosphate buffer and applied for biosensor incubation as described above. The recovery was assessed using the calibration plot obtained with a standard drug solution in pure buffer. For the three nominal concentrations of doxorubicin (10, 1 pM and 1.0 nM), average recovery was equal to 105 ± 7% for doxorubicin-TEVA and 102 ± 10% for doxorubicin-LANS; thus, the stabilizers used in these medicines (lactose and mannite, respectively) did not interfere with the doxorubicin measurement.

## 4. Discussion

The AY adsorption on the CB-covered GCE considered in this work offered a new mechanism of DNA intercalator determination. It is based on the influence of an analyte on the aggregation of the dye on the electrode affected by DNA and governed by the charge distribution on the electrode interface. As was shown by SEM and EIS, consecutive incubation of the GCE in reactant solutions resulted in their adsorption on the electrode surface due to alternate charge of the species (negatively charged CB particles and DNA molecules and positively charged AY molecules). Due to hydrophobicity of the AY molecules and their low solubility in water, adsorption of the dyes resulted in the formation of aggregates which did not exert remarkable redox activity. To some extent, the problem was solved by the treatment of the layer with DMF that partially dissolved the AY aggregates and promoted a more even distribution of the dye in the layer. The DNA molecules adsorbed promoted the disaggregation of the dye due to their interaction with phosphate groups of the DNA helix. Thus, intercalation partially compensated for the charge of native DNA, which decreased this effect and showed lower AY peak currents.

The DNA sensor developed made it possible to determine low concentrations of doxorubicin as a model intercalator in pico- and nanomolar ranges of its concentrations. No significant interference of sulfonamide drugs (sulfamethoxazole), serum proteins (bovine serum albumin) or plasma electrolytes (Ringer–Locke solution) with the doxorubicin determination was found. The DNA sensor was also tested for the determination of doxorubicin in two medications with satisfactory recovery and no influence of stabilizers (lactose and mannite). Although the medical application of the DNA sensor developed requires much more attention to the measurement precision and sample matrix effects, the concept of the DNA sensor assembling with no electropolymerization steps and variation of the sensitivity by treatment with organic solvent appears attractive for further development of similar DNA sensors for medical diagnostics and pharmacokinetics monitoring.

## Figures and Tables

**Table 1:** EIS parameters corresponded to various steps of the surface layer assembling (average ± S.D. for five electrodes).

Layer Content	Electrode–Film Interface			Film–Solution Interface		
	*R*_1_, kΩ	*C*_1_, μF	*n*_1_	*R*_2_, kΩ	*C*_2_, μF	*n*_2_
CB	0.224 ± 0.010	3.60 ± 0.20	0.990	1.71 ± 0.11	52.3 ± 1.42	0.993
CB/AY	0.290 ± 0.015	2.30 ± 0.22	0.998	2.25 ± 0.15	19.3 ± 0.55	0.997
CB/AY, DNA adsorption	0.480 ± 0.028	0.42 ± 0.18	0.998	4.53 ± 0.26	4.86 ± 1.12	0.997

**Table 2:** Analytical characteristics of the determination of doxorubicin with electrochemical sensors and DNA sensors.

Electrode Modifier/Electrode	Concentration Range, μM	Limit of Detection, nM	Ref.
Electrochemical sensors			
Multiwalled carbon nanotubes/GC	0.02–0.5	-	[48]
ZnO dispersed in graphite paste	0.07–5000	9.0	[49]
Composite of mesoporous carbon nanospheres and reduced graphene oxide/GCE	0.01–10	1.5	[50]
Pyrographite	0.01–1.0	10	[51]
Silver solid amalgam	0.6–10	440	[52]
Electrochemical DNA sensors			
Electropolymerized Azure B	0.0001–0.1	0.07	[36]
Electropolymerized aniline	1 × 10^−6^–1000	0.0006	[53]
Carbon nanotubes/poly(L-lysine) composite	0.0025–0.25	1.0	[54]
Electropolymerized Neutral red	0.01–100	0.1	[55]
Single-walled carbon nanotubes	0.001–20	0.6	[56]
Polyelectrolyte complexes with poly(styrene sulfonate) and aminated thiacalix[4]arene	0.1–100	0.1	[57]
AY and DNA adsorbed on CB/GCE	1 × 10^−5^–0.001	0.0007	This work

## Supplementary Materials

The following are available online at https://www.mdpi.com/article/10.3390/s21227763/s1, Figure S1: The dependence of the cathodic peak current of 1.0 μM AY in 0.025 phosphate buffer + 0.1 M NaNO_3_, pH = 7.0, on the scan rate (a) and square root from the scan rate (b). Average from six replications; Figure S2: The pH dependence of the cathodic peak current of 1.0 μM AY in 0.025 phosphate buffer + 0.1 M NaNO_3_, pH = 7.0, 100 mV/s. Average from six replications; Figure S3: Cyclic voltammograms recorded on GCE covered with the CB/AY mixture from the phosphate buffer. The surface layer content is expressed as v:v ratio of the components mixed prior to deposition on bare GCE. 1—5:1, 2—10:1, 3—1:1, 4—1:5, 5—1:10 (the numbers correspond to appropriate lines of the Table S1); Figure S4: (a,c) Cyclic voltammograms and (b,d) the anodic AY peak currents recorded on GCE modified with the CB/AY (1:1) + DNA (15 min incubation) after the DMF treatment (20 min). The AY concentration: 1–0.9 mM; 2–1.8 mM; (3–7)–3.6 mM; DNA concentration: 1–3, 6–1 mg/mL, 4–0 mg/mL, 5–0.2 mg/mL, 7–2 mg/mL, *n* = 5; Figure S5: The dependence of the anodic AY peak currents on the period of incubation of the CB/AY (1:1) modified GCE in DNA solution. Measurements in 0.025 M phosphate buffer + 0.1 M NaNO_3_, 100 mV/s, *n* = 5. Table S1: Volume ratio and final concentrations of the components in the mixtures of the stock solutions of the carbon black and AY applied for the electrode modification.
