# Supplementary material for: Electrochemical DNA Sensor Based on Acridine Yellow Adsorbed on Glassy Carbon Electrode
Source: Sensors (Basel). 2021 Nov 22;21(22):7763. doi: 10.3390/s21227763 (PMC8621912; doi:10.3390/s21227763)
Supplement: Supplementary file 1 [file sensors-21-07763-s001.zip › sensors-1473798-supplementary.pdf]

Electronic Supporting Information

to the article of Tatjana Kulikova, Anna Porfireva, Alexey Rogov and Gennady Evtugyn "Electrochemical DNA Sensor based on Acridine Yellow Adsorbed on Glassy Carbon Electrode"

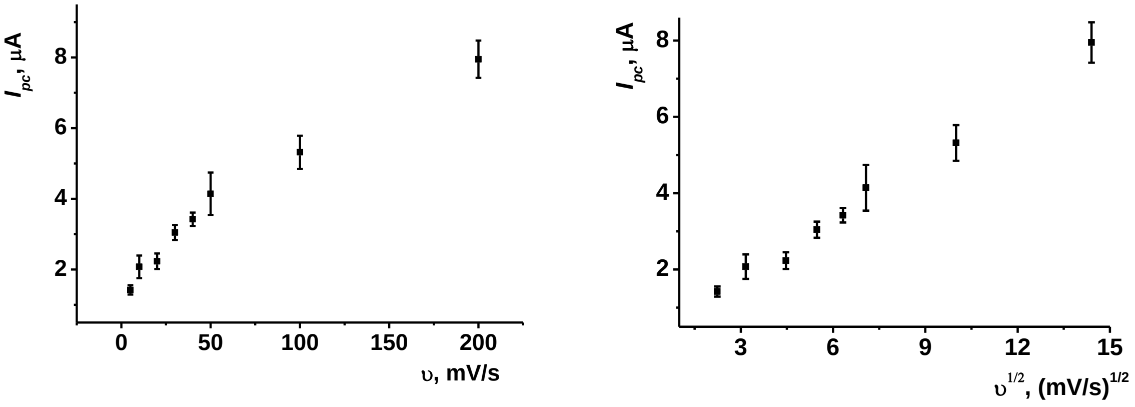

(a) **(b)**

Figure S1. The dependence of the cathodic peak current of 1.0 µM AY in 0.025 phosphate buffer + 0.1 M $NaNO_3$, pH = 7.0, on the scan rate (a) and square root from the scan rate (b). Average from six replications

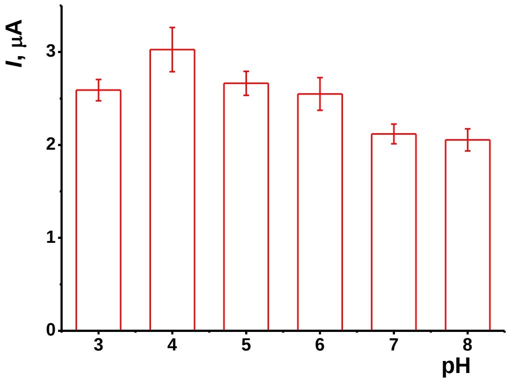

Figure S2. The pH dependence of the cathodic peak current of 1.0 µM AY in 0.025 phosphate buffer + 0.1 M $NaNO_3$, pH = 7.0, 100 mV/s. Average from six replications

Table S1. Volume ratio and final concentrations of the components in the mixtures of the stock solutions of the carbon black and AY applied for the electrode modification

No	Volume ratio carbon black : AY	Carbon black suspension, mg/mL	AY, mM
1	1:1	0.05	1.83
2	1:5	0.017	3.03
3	1:10	0.01	3.32
4	5:1	0.08	0.62
5	10:1	0.09	0.33

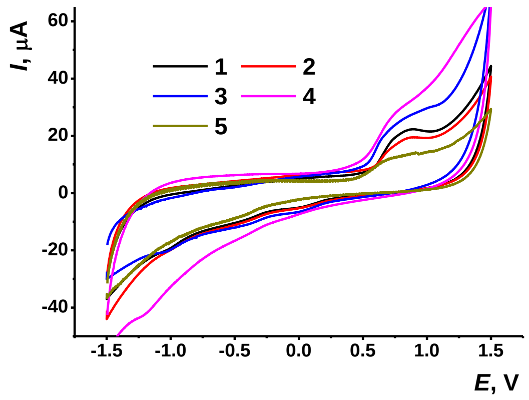

Figure S3. Cyclic voltammograms recorded on GCE covered with the CB/AY mixture from the phosphate buffer. The surface layer content is expressed as v : v ratio of the components mixed prior to deposition on bare GCE. 1 - 5 : 1, 2 - 10 : 1, 3 - 1 : 1, 4 - 1 : 5, 5 - 1 : 10 (the numbers correspond to appropriate lines of the Table S1)

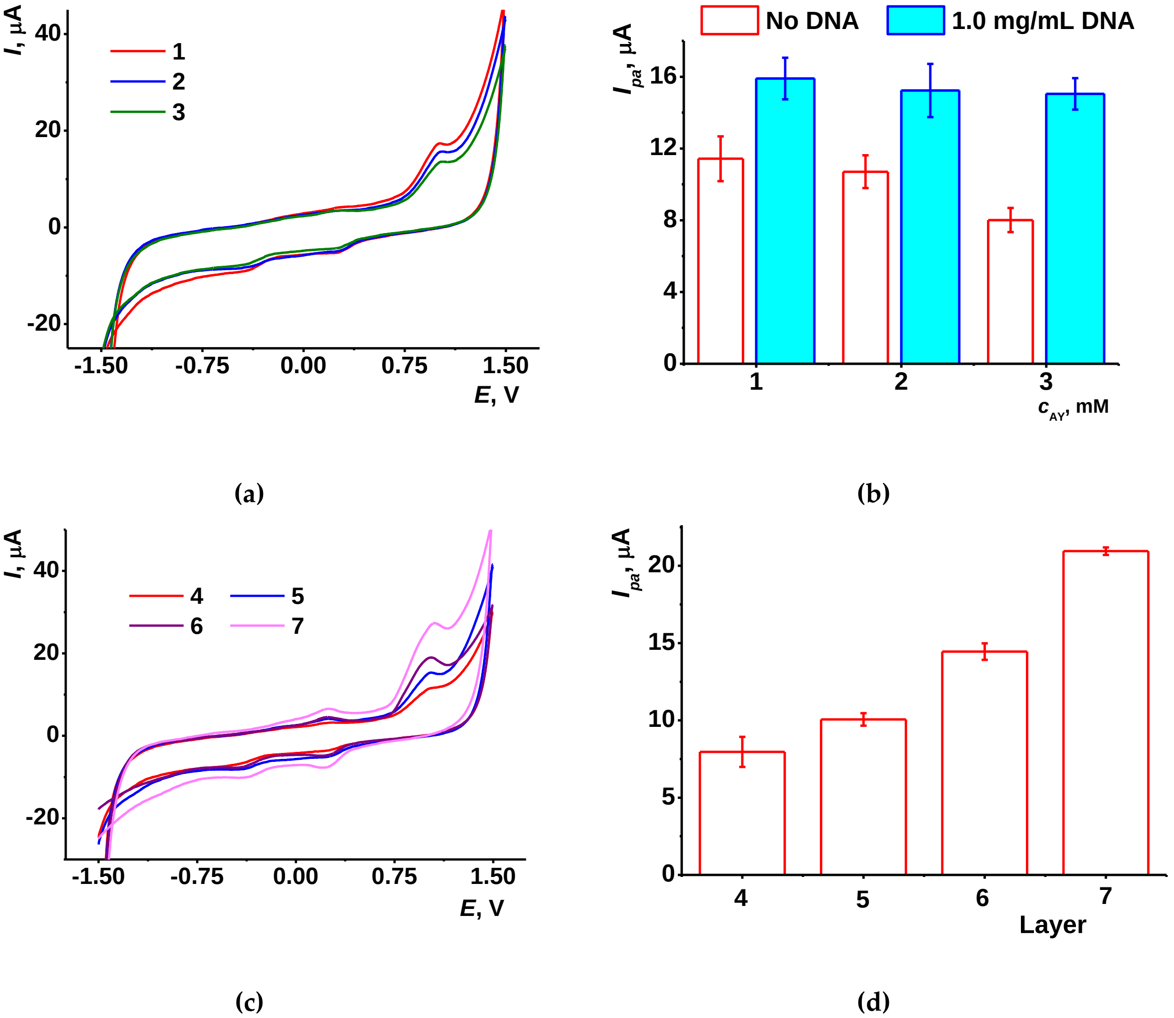

Figure S4. (a, c) Cyclic voltammograms and (b, d) the anodic AY peak currents recorded on GCE modified with the CB / AY (1:1) + DNA (15 min incubation) after the DMF treatment (20 min). The AY concentration: 1 - 0.9 mM; 2 – 1.8 mM; (3-7) – 3.6 mM; DNA concentration: 1-3, 6 – 1 mg/mL, 4 – 0 mg/mL, 5 – 0.2 mg/mL, 7 – 2 mg/mL, n=5

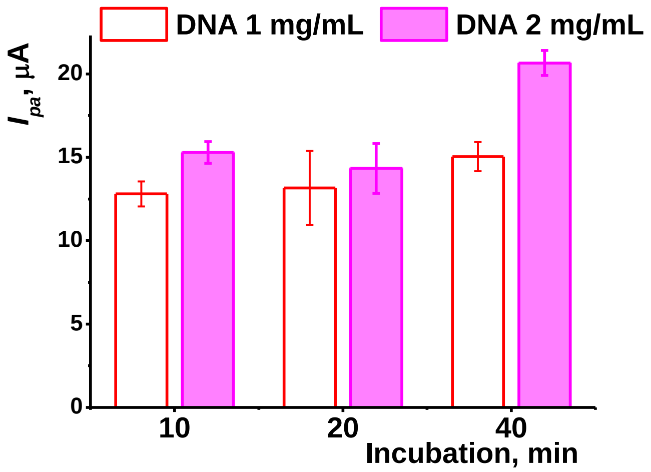

Figure S5. The dependence of the anodic AY peak currents on the period of incubation of the CB/ AY (1:1) modified GCE in DNA solution. Measurements in 0.025 M phosphate buffer + 0.1 M $NaNO_3$, 100 mV/s, n = 5.
